# Phenotypic Analysis of Diseased Plant Leaves Using Supervised and Weakly Supervised Deep Learning

**DOI:** 10.34133/plantphenomics.0022

**Published:** 2023-01-16

**Authors:** Lei Zhou, Qinlin Xiao, Mohanmed Farag Taha, Chengjia Xu, Chu Zhang

**Affiliations:** ^1^ College of Mechanical and Electronic Engineering, Nanjing Forestry University, Nanjing, China.; ^2^ College of Biosystems Engineering and Food Science, Zhejiang University, Zhejiang, China.; ^3^ Department of Soil and Water Sciences, Faculty of Environmental Agricultural Sciences, Arish University, North Sinai 45516, Egypt.; ^4^ School of Information Engineering, Huzhou University, Huzhou, China.

## Abstract

Deep learning and computer vision have become emerging tools for diseased plant phenotyping. Most previous studies focused on image-level disease classification. In this paper, pixel-level phenotypic feature (the distribution of spot) was analyzed by deep learning. Primarily, a diseased leaf dataset was collected and the corresponding pixel-level annotation was contributed. A dataset of apple leaves samples was used for training and optimization. Another set of grape and strawberry leaf samples was used as an extra testing dataset. Then, supervised convolutional neural networks were adopted for semantic segmentation. Moreover, the possibility of weakly supervised models for disease spot segmentation was also explored. Grad-CAM combined with ResNet-50 (ResNet-CAM), and that combined with a few-shot pretrained U-Net classifier for weakly supervised leaf spot segmentation (WSLSS), was designed. They were trained using image-level annotations (healthy versus diseased) to reduce the cost of annotation work. Results showed that the supervised DeepLab achieved the best performance (IoU = 0.829) on the apple leaf dataset. The weakly supervised WSLSS achieved an IoU of 0.434. When processing the extra testing dataset, WSLSS realized the best IoU of 0.511, which was even higher than fully supervised DeepLab (IoU = 0.458). Although there was a certain gap in IoU between the supervised models and weakly supervised ones, WSLSS showed stronger generalization ability than supervised models when processing the disease types not involved in the training procedure. Furthermore, the contributed dataset in this paper could help researchers get a quick start on designing their new segmentation methods in future studies.

## Introduction

Plant disease is one of the major threats that substantially impact the fruit yield and quality of plants [1,2]. They are caused by fungi, virus-carrying pests, and other stressors in the surrounding environment of plant growth, which damages plant health to varying degrees. Accurate and efficient detection of plant diseases is of great importance to help the producers take plant protection measures in time.

Conventional plant disease monitoring relies on human experts or simple color feature analysis methods. The former requires adequate experience and knowledge, which is always of high cost. The latter could only handle images with a simple background and be hard to ensure its robustness, such as extra-green (ExG) feature segmentation and threshold segmentation [3]. Recently, a fusion of emerging technologies including deep learning [4], computer vision [5], and spectroscopy [6,7] has been widely studied for plant phenotyping. The basic application of plant disease analysis is binary classification, which aims at dividing the images into a healthy group and a diseased group [8]. Then, more challenging tasks were proposed. Images of different leaves, diseases, and degrees of infection were expected to be identified. Famous public datasets, such as Plant Village (https://arxiv.org/abs/1511.08060), and some of its improved versions gained attention of many researchers, which led to the emergence of new ideas for disease classification [9]. With the demand for precision plant disease management, advanced tasks like localization of the disease symptom, disease spots distribution analysis, and other phenotypic feature extraction need more attention. Successful cases could be found, including disease-damaged leaf detection [10] and lesion segmentation [11].

Deep learning methods have many advantages to be considered a powerful tool for plant disease segmentation. However, the training procedure of the convolutional neural network (CNN) segmentation models requires adequate samples with pixel-level annotations. The cost of time and manual labor is high for pixel-level annotation making. To our knowledge, most of the public datasets only provided image-level annotation for classification studies. There are very few published with pixel-level annotations.

Weakly supervised learning, a novel idea that attempts to use training samples that are not sufficiently annotated, has become a trending topic in recent years [12,13]. When used for segmentation purposes, such methods generate attention maps, saliency maps [14], explaination maps [15], or class activation maps (CAM) [12,16,17] from the intermediate features related to the target class to determine the locations of the objects assigned with the same class index. With weakly supervised learning methods, the CNN models should be informed of what objects are involved in the image, and then it can output where the objects are. The advantages of such methods could be found in the cost of the annotation.

This study conducted experiments on diseased leaf phenotyping based on deep learning and computer vision. A plant leaf dataset with both image-level and pixel-level annotations was presented. The DeepLab model and U-Net model under supervised training were established as the baseline. Inspired by these novel ideas of weakly supervised learning, the possibility of CNN models trained using image-level annotation to realize disease spot segmentation was explored.

## Materials and Methods

### Dataset

In this study, the RGB images of plant leaves were collected from several datasets published on the Internet. The annotations for training and evaluating semantic segmentation models were prepared by the authors. We uploaded the images and the corresponding annotation to the Mendeley Data repository (https://data.mendeley.com/datasets/tsfxgsp3z6). The dataset is also available at https://pan.baidu.com/s/1y7K2dVpfkQ3HVOU1qEeChQ (password: ecff). The details about the dataset preparation are introduced below.

#### Images

The color images were collected from public datasets, including a popular open access dataset called Plant Village (available at https://data.mendeley.com/datasets/tywbtsjrjv/1) and a dataset of diseased apple leaves (available at https://aistudio.baidu.com/aistudio/datasetdetail/11591). The Plant Village dataset includes 14 crop varieties and 26 diseases, and the diseased apple leaves dataset includes 4 kinds of apple disease. Both of them were established for disease type classification purposes. In this research, we selected a part of plant disease images with spot symptoms for study. To unify the format of images from different sources, the images in the diseased apple leaves dataset were resized to 256 × 256 resolution, and those in the Plant Village dataset were augmented by rotation, flipping, brightness, and contrast value adjustment.

#### Annotations

The annotations were prepared for training the supervised segmentation networks and evaluating the performances of the weakly supervised models. A free image editing software called Paint.net (available at https://www.getpaint.net/) was used for preparing the pixel-level annotation. First, an image is opened by the software, and a new layer is added as the top layer. Second, a pencil with a line width of 1 pixel is used to depict the edge of a spot to form a closed area. Next, the closed areas are filled with the same color as the edge line. Finally, only the top layer is set as visible and saved as an annotation file. Details of these steps are shown in the Supplementary Materials.

The information about the studied disease type and the number of annotated images are listed in Table 1. All listed samples with apple leaf diseases were used for training, optimization, and evaluation of the supervised and weakly supervised learning models. As for the configuration of the datasets used for classification and segmentation, a ratio of 6:2:2 was used to divide the samples into a training dataset, a validation dataset, and a testing dataset. The rest part (i.e., the images of grape leaf disease and strawberry leaf disease) were defined as an extra testing dataset and used to check the generalization ability of the models when handling the disease types and leaf types not involved in the training and optimization procedure.

**Table 1.** Disease types and the number of samples.

Type	Disease name	Source	Number of images with pixel-level annotation	Number of images with image-level annotation
Healthy apple leaves	/	Plant Village dataset	/	21,385
Diseased apple leaves	Apple scab	Plant Village dataset	2,028	24,507
	Black rot	Plant Village dataset	2,119	
	Cedar apple rust	Plant Village dataset	1,247	
	Alternaria boltch	Diseased apple leaves dataset	1,820	
	Rust	Diseased apple leaves dataset	4,056	
Other diseased leaves	Grape—Black rot	Plant Village dataset	1,300	/
	Grape—Esca (black measles)	Plant Village dataset	1,300	
	Strawberry—Leaf scorch	Plant Village dataset	1,209	

### Diseased spot segmentation methods

#### Supervised CNNs for segmentation

**Fig. 1.**
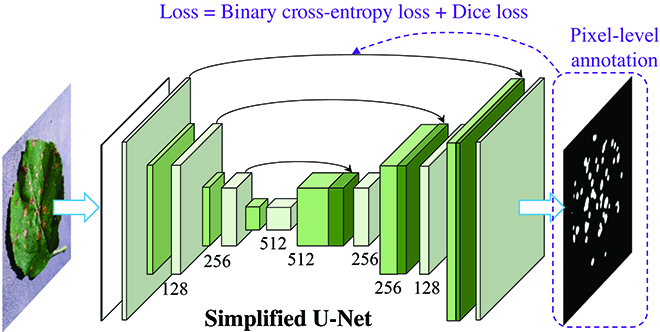
The architecture of the simplified U-Net for disease spot segmentation. DeepLab models [30] are also well-known CNN-based models for image segmentation. The integrated dilated convolution units help these models reach higher performances. They were applied for the segmentation of leaves or plants under complex scenarios [30,31].

U-Net is a popular deep learning network for semantic segmentation, which is famous in medical image segmentation applications [18]. Currently, there are also quite a few successful cases in plant phenotyping achieved by the U-Net, such as separating plants from the background [19] and plant root segmentation [20]. In this study, a simplified U-Net model was adopted for leaf disease spot segmentation. The most remarkable characteristic of this model is the use of skip connections. It transmits the features of shallow convolution layers (with rich low-level information) to the high-level convolution layers, which is more conducive to generating segmentation masks. Its overall structure is shown in Fig. 1.

For convenience, the supervised learning-based U-Net and DeepLab models are defined as U-Net-S and DeepLab-S, respectively. For training the supervised segmentation CNNs, the initial learning rate was set as 0.0005, which was scheduled to be reduced to 1/10 after every 25 epochs. The maximum training epoch was defined as 100. The value of batch size was set according to the memory size of the GPU. The Adam [21] method was selected as the optimizer for training, targeting to minimize the fused loss function (binary cross-entropy loss and dice loss [22]) between the prediction and ground truth, which could be expressed as:

$$L_{\text{Dice}}=1-\frac{2\left|y_{p}\right|\cdot \left|y\right|+\varepsilon }{\left|y_{p}\right|+\left|y\right|+\varepsilon }$$ (1)

$$\begin{array}{c} L_{\mathrm{BCE}}=-\left\{y_{p}\cdot \text{log}\sigma \left(y\right)\right.\left.+\left(1-y_{p}\right)\cdot \text{log}\left[1-\sigma \left(y\right)\right]\right\} \end{array}$$ (2)

$$L=\lambda L_{\mathrm{BCE}}+\left(1-\lambda \right)L_{\text{Dice}}$$ (3)

where *L_Dice_* denotes the dice loss, *L_BCE_* is the loss of binary cross-entropy with logits, *y_p_* and *y* are the predicted value and ground truth, *ε* is a small value to ensure that the denominator is a non-zero value, *σ* is the sigmoid function, *λ* is the weight of *L_BCE_*, and *L* is the final loss function of the segmentation models. *λ* was set as 0.5 in this study.

For each training epoch, the training loss was calculated for parameter tuning, while the validation loss was used for model evaluation and optimized parameter selection. At the end of each epoch, if the new validation loss was lower than that in the previous epoch, the current status of the model (weights and bias of the CNN model) will be saved to overwrite the previously saved one. Therefore, the best model that produced the lowest validation loss was preserved.

#### Weakly supervised methods for segmentation

The mentioned U-Net and DeepLab models require a supervised learning procedure. The ground truth for model training is a mask (pixel-level annotation) with the same width and height as the image to be segmented. In other words, the models need to know where the disease spot is during the supervised learning-based training procedure. In this study, weakly supervised disease spot segmentation methods were explored, which need to be informed whether the leaves are healthy or not (image-level annotation) during the model training. Then, the trained model could discriminate the lesion area and realize semantic segmentation.

Grad-CAM [23] is a very popular method that extracts the visual evidence for the CNN image classifier. It makes the abstracted deep learning model explainable by generating a CAM using the diseased class-related weights in the output layer and the feature maps before the global average pooling layer. The generated CAM could be considered as a heat map, in which higher values indicate stronger attention on the corresponding area. Afterward, this method has been applied for weakly supervised semantic segmentation. Some necessary parts are required for establishing weakly supervised segmentation methods. The first part (P1) is to establish a binary classifier, forcing the extracted features to represent the basis for distinguishing healthy and diseased leaves. The second part (P2) should be the generation of a rough distribution map of disease symptoms. The third part (P3) should be designed to refine the rough distribution map to get a more precise mask as the final segmentation result. Inspired by the characteristics of Grad-CAM, 2 kinds of weakly supervised CNN models were designed in this research.

**Fig. 2.**
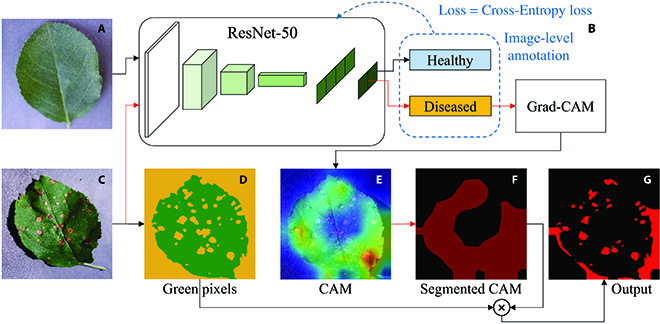
The combination of ResNet and Grad-CAM for weakly supervised segmentation. (A) Image of a healthy leaf. (B) Classification model and Grad-CAM. (C) Image of a diseased leaf. (D) Extra-green feature segmentation result; green area denotes the green pixels, and yellow area denotes the non-green pixels. (E) Output of Grad-CAM. (F) Segmented activated map; dark red area denotes the foreground of the activation map. (G) Final output of the pseudo-mask; red area is the foreground of the pseudo-mask.

The first one was the combination of a ResNet-50 classification model and the Grad-CAM [23] method, which was defined as ResNet-CAM (see Fig. 2). The steps for establishing the ResNet-CAM model included (1) a binary classification ResNet-50 model that was trained using image-level annotation (healthy versus diseased) and the cross-entropy loss. The learning rate was 0.0005. The initial parameters were transferred from the ImageNet. The model with the lowest validation loss was saved. (2) A feature map with the same size as the input image was generated by the Grad-CAM method. Another method named saliency map [14] could also generate an attention map to indicate the important areas with strong attention. It would be compared with the Grad-CAM method for the qualities of the generated feature maps. (3) The feature map was processed by adaptive threshold segmentation and modified extra-green feature segmentation to establish a pseudo-mask, which was regarded as the final segmentation result. The calculation of the modified extra-green feature could be expressed as:

$${\text{Mask}}_{\text{green}}=\left(2G-R-B\right)>T\&\left(G>R\right)\&\left(G>B\right)$$ (4)

where *Mask_green_* is a matrix indicating the distribution of the green pixels. R, G, and B denote the red, green, and blue channels, respectively. *T* is a threshold, which could be determined by the Ostu algorithm [24]. The pseudo-mask shown in Fig. 2G was the intersection of the yellow area in Fig. 2D and dark red area in Fig. 2F. (4) An optional step, the generated pseudo-masks could be further used for training a semantic segmentation model and improving the accuracy. Overall, P1 involves step 1, P2 involves step 2, and P3 involves steps 3 and 4 of ResNet-CAM.

**Fig. 3.**
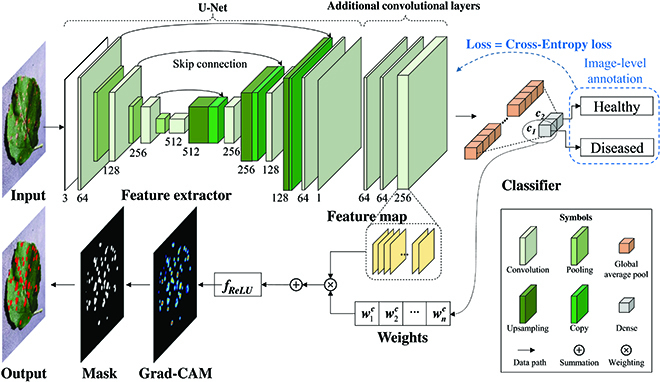
The overall structure of the WSLSS method.

The second solution combined a few-shot pretrained feature extractor, a binary classifier, and the Grad-CAM method for weakly supervised leaf spot segmentation, which was defined as WSLSS. Its architecture is illustrated in Fig. 3. (1) A simplified U-Net was pretrained using few-shot learning, in which only a small number of pixel-level annotations were employed for supervision. (2) Three convolution layers, a global average pooling layer, and a dense layer were added to construct a binary classifier. (3) This pretrained classifier was fine-tuned using a large dataset that only contained image-level annotation. (4) A CAM was calculated by the Grad-CAM method. (5) The final segmentation result was generated by processing the CAM using adaptive threshold segmentation and extra-green segmentation. For the WSLSS method, P1 involves steps 1, 2, and 3; P2 involves step 4; and P3 involves step 5.

The different numbers of training samples, including 200 and 400, were used to pretrain the backbone of the proposed WSLSS model. Then, WSLSS was fine-tuned using a similar training configuration to the previously mentioned ResNet-50 classifier. Specially, the WSLSS model used a smaller initial learning rate of 0.00005 for fine-tuning, to remain the knowledge from the pretraining task.

#### Performance metrics, hardware, and software

The CNN models were trained and evaluated using the dataset of apple leaves samples. Furthermore, all prepared images of diseased grape leaves and diseased strawberry leaves were used as an extra testing dataset for checking the generalization ability of the studied models. The performances of segmentation methods are commonly evaluated by Intersection over Union (IoU), Precision, and Recall. IoU calculates the proportion of corrected identified pixels in the union of predicted and true disease pixels. Precision calculates the ratio of correctly detected diseased pixels to the pixels predicted as positive, and Recall indicates the ratio of correctly detected diseased pixels to the true diseased pixels. The calculations of them are provided as Eqs. 5 to 7:

$$\mathrm{IoU}=\frac{\mathrm{TP}}{\mathrm{TP}+\mathrm{FP}+\mathrm{FN}}$$ (5)

$$\text{Precision}=\frac{\mathrm{TP}}{\mathrm{TP}+\mathrm{FP}}$$ (6)

$$\text{Recall}=\frac{\mathrm{TP}}{\mathrm{TP}+\mathrm{FN}}$$ (7)

where *TP* denotes true-positive pixels, *FP* denotes false-positive pixels, and *FN* denotes false-negative pixels. IoU, Precision, and Recall of the whole dataset were calculated by averaging the image-level performance metrics.

The segmentation outputs of these mentioned methods were saved as Portable Network Graphics format (.png) for performance metrics calculation and comparison. In addition, the outputs of the intermediate steps were also visualized for analysis.

The deep learning-based image processing tasks were conducted on a desktop PC, with Intel Core i7-8700K CPU, 64GB DDR4 RAM, 512 GB SSD, and an NVidia RTX 3090 GPU (24 GB). The PC is installed with Ubuntu 20.04, python 3.6, PyTorch, and other necessary modules for running deep learning codes.

## Results

**Fig. 4.**
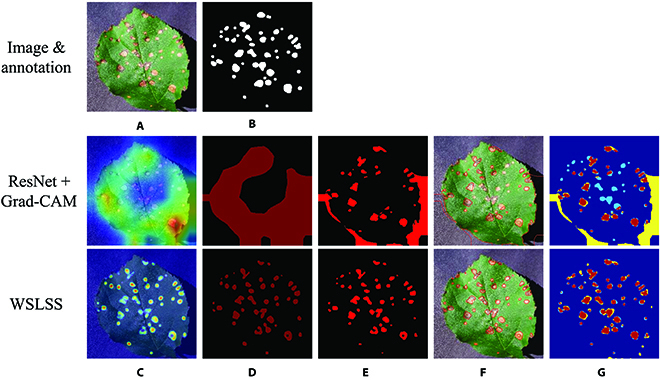
Comparison between the results achieved by ResNet-CAM and WSLSS. (A) RGB image. (B) Annotation. (C) CAM; the colormap covered on the original image denotes the attention weight values. (D) Segmented CAM; dark red denotes the foreground, and black denotes the background. (E) Final segmentation result. (F) Edge lines of the detected spots. (G) Ground truth versus prediction; red denotes true positive, yellow denotes false positive, light blue denotes false negative, and dark blue denotes true negative.

First, the segmentation performances on the diseased apple leaves were analyzed. The binary classifiers used in the weakly supervised segmentation methods achieved satisfactory classification accuracies (>98.5%). Therefore, the classification results would not be further discussed here. Table 2 lists the final leaf spot segmentation results. Figure 4 provides several samples of segmentation results achieved by the studied weakly supervised models. It could be observed that U-Net-S and DeepLab-S achieved very high performances, which significantly outperformed the studied weakly supervised models including ResNet-CAM and WSLSS. The best performance was achieved by DeepLab-S, with IoU = 0.829, Precision = 0.897, and Recall = 0.905 on prediction dataset. As for weakly supervised models, the typical method, which was defined as DeepLab+Pseudo (a semantic segmentation model trained using the pseudo-label generated by Grad-CAM), achieved an 0.190 of IoU, 0.287 of AP, and 0.505 of AR on the testing dataset. Without the pseudo-label-based retraining and ExG method, the IoU values of those methods were even lower. The highest performance of weakly supervised models was produced by WSLSS, with IoU = 0.434, Precision = 0.747, and Recall = 0.585. When comparing the qualities of feature maps provided by Grad-CAM and saliency map, it could be observed that the saliency map method produced a low IoU of 0.03. Therefore, saliency map-based segmentation methods would not be further studied in this study.

**Table 2.** Performance metrics of the CNN models on apple leaf disease spot segmentation.

Training mode	Model	Dataset	IoU	Precision	Recall
Supervised learning	U-Net-S	Training	0.799	0.884	0.884
		Validation	0.745	0.850	0.849
		Testing	0.750	0.854	0.854
	DeepLab-S	Training	0.914	0.944	0.962
		Validation	0.827	0.896	0.903
		Testing	0.829	0.897	0.905
Weakly supervised learning ^a^	Saliency Map	Training	0.031	0.326	0.037
		Validation	0.031	0.323	0.038
		Testing	0.030	0.320	0.035
	Simple Grad-CAM ^b^	Training	0.108	0.109	0.930
		Validation	0.107	0.108	0.930
		Testing	0.107	0.108	0.933
	Grad-CAM + ExG ^c^	Training	0.182	0.283	0.489
		Validation	0.180	0.278	0.491
		Testing	0.182	0.280	0.489
	DeepLab + Pseudo ^d^	Training	0.188	0.289	0.499
		Validation	0.187	0.285	0.504
		Testing	0.190	0.287	0.505
	DeepLab +Pseudo + ExG ^e^	Training	0.197	0.308	0.497
		Validation	0.194	0.301	0.501
		Testing	0.198	0.304	0.503
Weekly supervised learning with few-shot pixel-level annotation	WSLSS-200 ^f^	Training	0.357	0.713	0.511
		Validation	0.351	0.711	0.504
		Testing	0.356	0.708	0.514
	WSLSS-400 ^g^	Training	0.431	0.749	0.579
		Validation	0.430	0.745	0.581
		Testing	0.434	0.747	0.585

^a^ DeepLab + Pseudo + ExG denotes the complete ResNet-CAM method. Simple Grad-CAM, Grad-CAM + ExG, and DeepLab + Pseudo are the methods realized by removing some parts of ResNet-CAM.

^b^ The direct output from the Grad-CAM method.

^c^ The output is processed by Grad-CAM and ExG segmentation.

^d^ DeepLab trained with the pseudo-annotation generated by Grad-CAM.

^e^ DeepLab trained with the pseudo-annotation generated by Grad-CAM and ExG segmentation.

^f^ WSLSS pretrained using 150 and 50 samples for training and validation, respectively.

^g^ WSLSS pretrained using 300 and 100 samples for training and validation, respectively.

In this study, ablation experiments were conducted to check the effects of each module on the overall performance of the ResNet-based segmentation methods. The results can be found in Table 2. When considering the CAM (the direct output of the Grad-CAM method) as the final segmentation result, the IoU was 0.107. Then, the rough segmentation was refined by the modified ExG segmentation method, raising the IoU to 0.182. Furthermore, the rough segmentations and the refined ones were used as pseudo-annotations to train DeepLab models. However, the supervised model trained using pseudo-labels did not get significant improvement on the final performance (IoU < 0.2).

The pictures in Fig. 4 show the intermediate output features of the Grad-CAM and ExG segmentation. As shown in Fig. 4C and D, the method ResNet-CAM could detect the areas containing spots. However, the detected distribution map was relatively rough. A certain number of background (shadow) pixels were included, and some of the diseased areas were not covered, which led to a low IoU. On the other hand, the WSLSS method performed better than the ResNet-CAM, providing a more accurate distribution map. Most of the pixels of the disease spots were highlighted and the shadows were not included in the segmentation result. In Fig. 4G, in the segmentation map provided by WSLSS, it can be observed that the red areas representing the correct segmentation occupy a large proportion.

Then, the models trained using apple leaves were further tested by the extra testing dataset, which consisted of images of grape leaves and strawberry leaves. For each type of model listed in Table 2, only the one with the best performance was evaluated by the extra dataset (see Table 3). It could be observed that the performances of supervised learning-based models were significantly reduced when processing the images with new leaf species and disease types. The IoU values of DeepLab-S and U-Net-S were lower than 0.5. On the other hand, weakly supervised models even reached higher accuracies on the extra testing dataset than those on the dataset only including apple leaves. The generalization ability of WSLSS was encouraging. Its IoU value on the extra testing dataset was 0.511, which was even better than that on the apple leaf dataset.

**Table 3.** Performance metrics of the CNN models on the extra testing dataset.

Model	Dataset	IoU	Precision	Recall
U-Net-S	Extra testing dataset	0.437	0.472	0.860
DeepLab-S		0.458	0.501	0.846
ResNet-CAM		0.220	0.233	0.831
WSLSS		0.511	0.691	0.661

## Discussion

### The comparison between fully supervised and weakly supervised methods

According to Table 2, the results of weakly supervised segmentation were encouraging, though they were far from those realized by supervised methods when only evaluating using apple leaf samples. As a fact, such results were predictable because U-Net-S and DeepLab-S were trained with much informative knowledge (a large number of samples with pixel-level annotations), while the ResNet-CAM and WSLSS model were supported with weak labels (only know if the leaf in the image is healthy or diseased). A similar situation could be found in the research focusing on those non-agricultural datasets [25]. After all, supervised learning has obtained more accurate label information.

However, when testing the models using the extra dataset (samples of grape leaves and strawberry leaves; see Table 3), fully supervised models, including U-Net-S and Deeplab-S, performed worse than WSLSS. High accuracies on the apple leaf dataset limited the generalization ability of fully supervised models. To some extent, it could be regarded as overfitting. For the models trained using weak annotations, the variability of accuracy was relatively small when tested by different testing datasets. Therefore, according to the results in this research, weakly supervised learning-based models were relatively not sensitive to the involved disease types and leaf types in the training dataset. On this point, it has an advantage over the fully supervised CNN methods.

**Fig. 5.**
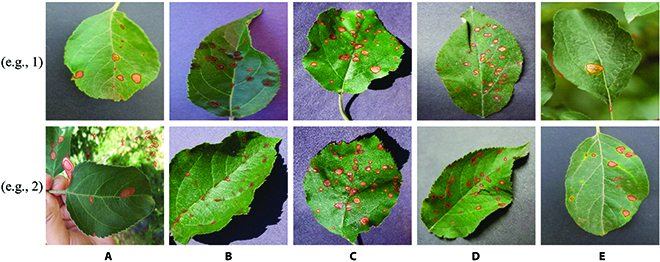
Segmentation examples of different kinds of apple leaf disease spots realized by WSLSS. (A) Alternaria boltch. (B) Apple scab. (C) Black rot. (D) Cedar apple rust. (E) Rust.

Figure 5 shows some WSLSS-segmented examples of different kinds of apple disease spots with relatively high performance. The WSLSS method performed better when detecting the spots with clear edges and simple background. When analyzing the leaves with apple scabs (see Fig. 5B), WSLSS could not completely cover all disease-infected areas due to the unclear symptom and the blurred edge line of such disease. Based on the limited annotation information, WSLSS is relatively hard to perfectly deal with the images with complicated backgrounds. The disease spot itself is always of small size. Even a small area of false-positive detected pixels would significantly reduce the value of IoU.

**Fig. 6.**
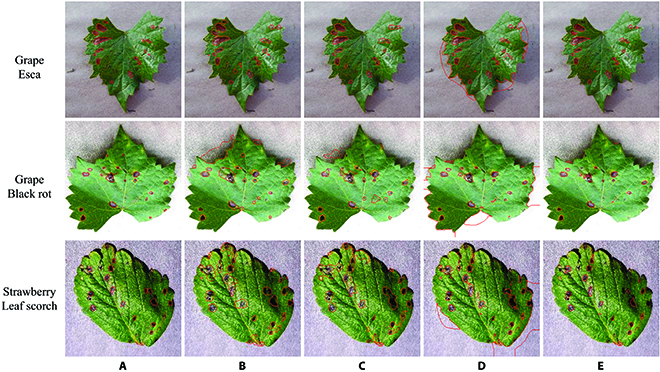
Segmentation examples of diseased grape and strawberry leaves realized by different CNNs. (A) Ground truth. (B) U-Net-S. (C) DeepLab-S. (D) ResNet-CAM. (E) WSLSS.

Figure 6 gives some segmentation examples from the extra testing dataset. The major problem of U-Net and DeepLab was oversegmentation, while the factor restricting segmentation accuracy of WSLSS was the undersegmentation problem.

The IoU value achieved by WSLSS on the extra testing dataset was 0.511, even better than that on the apple leaf dataset (IoU = 0.434). Possible reasons could be concluded as follows. The images in the extra testing dataset were all collected from the Plant Village dataset, with a relatively simple and clear background. Some image samples of the apple leaf dataset included complex backgrounds, such as the sample shown in Fig. 5A (e.g., 2) and Fig. 5E (e.g., 1).

### The effects of the individual modules in weakly supervised methods

Overall, the studied weakly supervised leaf disease spot segmentation methods included 3 parts. P1 establishes a CNN classifier, extracting the features for distinguishing healthy and diseased samples. P2 generates a raw distribution map of disease symptoms by Grad-CAM. P3 refines the raw distribution map to produce the final segmentation result based on adaptive threshold segmentation, extra-green feature segmentation, and pseudo-label-based semantic segmentation model retraining. The functions, advantages, and limitations of the modules in the mentioned parts would be discussed.

The classifier in P1 expects a classifier with high accuracy. The ResNet-50 model was selected in this study. Other famous models, such as the Vision Transformer (ViT) [26] and Swin Transformer (Swin-T) [27], were also tried. However, they produced a lower accuracy (<80%), which was suitable to process more complex tasks and required a much larger dataset for training. Hence, these models were not further discussed here. Moreover, it should be pointed out that the classifier consisting of segmentation models, extra convolution layers, and dense layers had a very large volume. It occupies a large amount of GPU memory during training, which has a negative impact on practical applications. Therefore, the DeepLab-based binary classifier was not explored. Instead, the U-Net-based model was selected.

As for P2, the Grad-CAM method provided a heat map representing the evidence for judging the image as the diseased category. As the example given in Fig. 4, the Grad-CAM method could effectively and accurately highlight the diseased area. However, it could be inferred that it was hard to completely cover all diseased areas. Actually, to accurately identify a diseased leaf, a small part of disease-related features, rather than complete disease areas on the leaf, is required. Hence, the features highlighted in Fig. 4C and D do provide adequate evidence for identifying the tested image as a diseased one, but not enough to extract all the diseased areas. As for another kind of weakly supervised learning-based application, objective counting [28], a small number of wrongly identified pixels or missed pixels will not significantly reduce the counting accuracy. Unlike the application for counting tasks [28], the evaluation of segmentation result in this study was relatively sensitive to correctly and wrongly identified pixels, which makes the weakly supervised segmentation tasks more challenging.

In P3, adaptive threshold segmentation was necessary for converting the feature map (float type) to a binary mask. The extra-green feature segmentation was effective in this research because the studied disease spots were orange or brown, or gray color. However, the single extra-green feature could not be applied for spot segmentation, because the background of the image was inseparable from the spots. Moreover, training a CNN segmentation model using pseudo-labels could slightly improve the IoU (0.182 to 0.198; see Table 2).

### Limitations and future perspectives

There are some specific problems in the disease spot segmentation task. The target areas were relatively small (see Figs. 4G and 6). Even a small number of incorrectly segmented pixels would significantly reduce the IoU value. However, the accuracy of the annotation is hard to control due to the indistinct edge of spots. In the explaination map-based study [15], the spatial correlation between annotations and predictions of a certain number of image samples was lower than 0.5, although the tested images had a very simple background. Moreover, the complex background of the image samples in this study also makes such tasks more challenging. The listed concerns in this paragraph become part of the reasons why the IoU of the leaf spot segmentation task seems not very high. On the contrary, in [29], the famous Pascal VOC dataset was analyzed, in which the objectives in an image are relatively large. Slight oversegmentation or undersegmentation problems would not significantly reduce the value of IoU.

The results showed that the highlighted area in the CAM sometimes covered the pixels that belong to the background, which also seriously affected the final segmentation performance. The key problem could be concluded as obtaining a more precise feature map. In future studies, the following solutions could be explored. (1) Preprocessing. For example, leaf object detection methods that effectively separate the leaf area from the background [10,27] could be used before the leaf spot segmentation step. (2) Refining the CAM by other computer vision methods, such as super-pixel segmentation. (3) Self-supervised ideas could be adopted to add constraints, making up for the lack of supervision information. The dataset with pixel-level annotations provided in this study can be a basis for researchers to conduct new segmentation experiments and to evaluate new methods in future studies.

## Conclusion

This study explored the application of deep learning for the phenotypic analysis of diseased plant leaves. Fully supervised and weakly supervised CNN models were established for disease spot segmentation. A diseased leaf image dataset with both image-level and pixel-level annotations was presented. When processing the dataset only involving apple leaves, the fully supervised DeepLab model reached the highest performance (IoU = 0.829). The WSLSS method achieved the best accuracy in weakly supervised models, with an IoU of 0.434. When these models were tested by the images with other plant species and diseases (grape diseases and strawberry diseases) that were not involved in the modeling training procedure, the performance of the fully supervised DeepLab model was significantly reduced, producing 0.458 of IoU. A slight change was observed in the performance metrics of WSLSS. It even reached the best IoU value of 0.511 in this study when processing the disease types not seen before, which revealed the robustness and generalization ability of weakly supervised learning-based CNNs. Besides these segmentation models, the published diseased images with annotated spot areas would help the researchers save much time on dataset preparation and have a quick start for exploring new deep learning methods for diseased leaf spot segmentation.

## Data Availability

The images and annotations used in this study were uploaded to a publicly available repository. The codes used in this study are available from the corresponding author upon reasonable request.
