# Erratum to “Phenotypic Analysis of Diseased Plant Leaves Using Supervised and Weakly Supervised Deep Learning”

**DOI:** 10.34133/plantphenomics.0033

**Published:** 2023-03-30

**Authors:** Lei Zhou, Qinlin Xiao, Mohamed Farag Taha, Chengjia Xu, Chu Zhang

**Affiliations:** ^1^ College of Mechanical and Electronic Engineering, Nanjing Forestry University, Nanjing, China.; ^2^ College of Biosystems Engineering and Food Science, Zhejiang University, Zhejiang, China.; ^3^ Department of Soil and Water Sciences, Faculty of Environmental Agricultural Sciences, Arish University, North Sinai 45516, Egypt.; ^4^ School of Information Engineering, Huzhou University, Huzhou, China.

In the Research Article “Phenotypic Analysis of Diseased Plant Leaves Using Supervised and Weakly Supervised Deep Learning,” one author’s name was published with a spelling error: Mohamed Farag Taha was listed as Mohanmed Farag Taha.

The legend of Fig. 1 included the paragraph “DeepLab models [30] are also well-known CNN-based models for image segmentation. The integrated dilated convolution units help these models reach higher performances. They were applied for the segmentation of leaves or plants under complex scenarios [30,31].” This paragraph was meant to be in the text of the article, after the paragraph “U-Net is a popular deep learning network for semantic segmentation,…” in Materials and Methods.

These errors were discovered by the authors after publication and do not affect the results, discussion, or conclusion of this paper. The author list, Fig. 1 figure legend, and Materials and Methods text have been corrected in the PDF and HTML (full text).
