# Supplementary figures and images for: Phenotypic Analysis of Diseased Plant Leaves Using Supervised and Weakly Supervised Deep Learning
Source: Plant Phenomics. 2023 Jan 16;5:0022. doi: 10.34133/plantphenomics.0022 (PMC10076051; doi:10.34133/plantphenomics.0022)

**Supplementary materials**


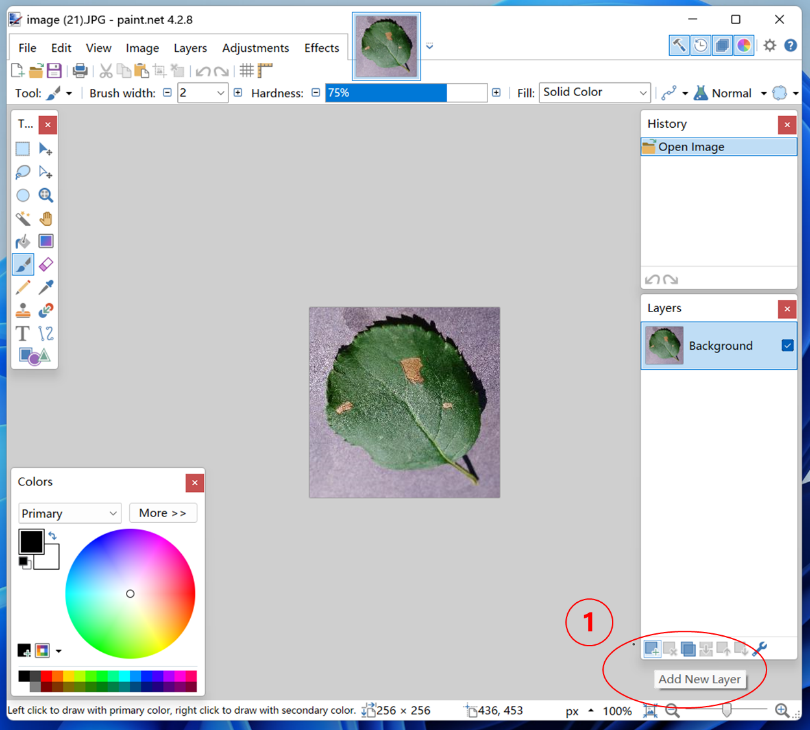

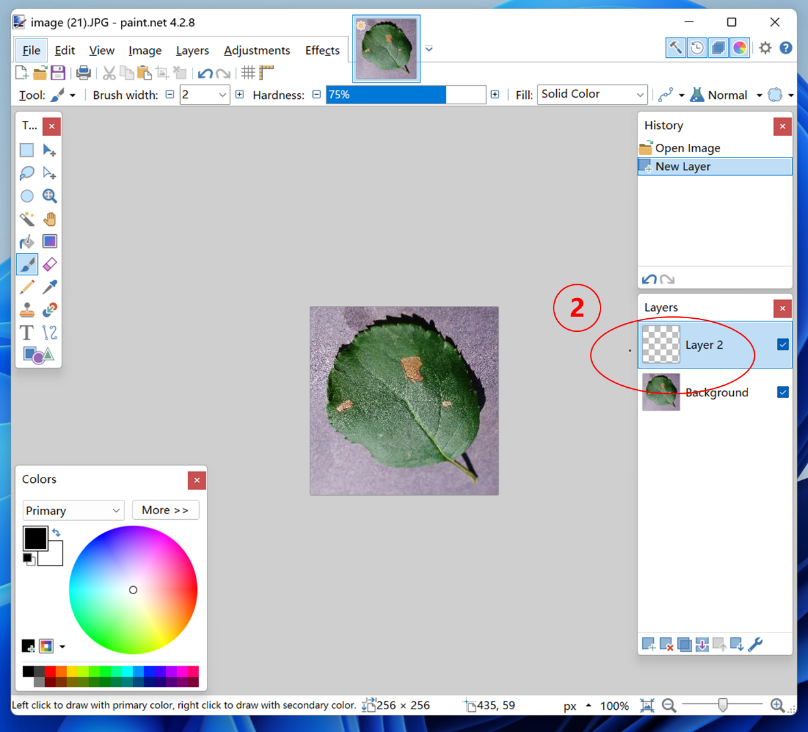


| (1) | (2) |
| --- | --- |


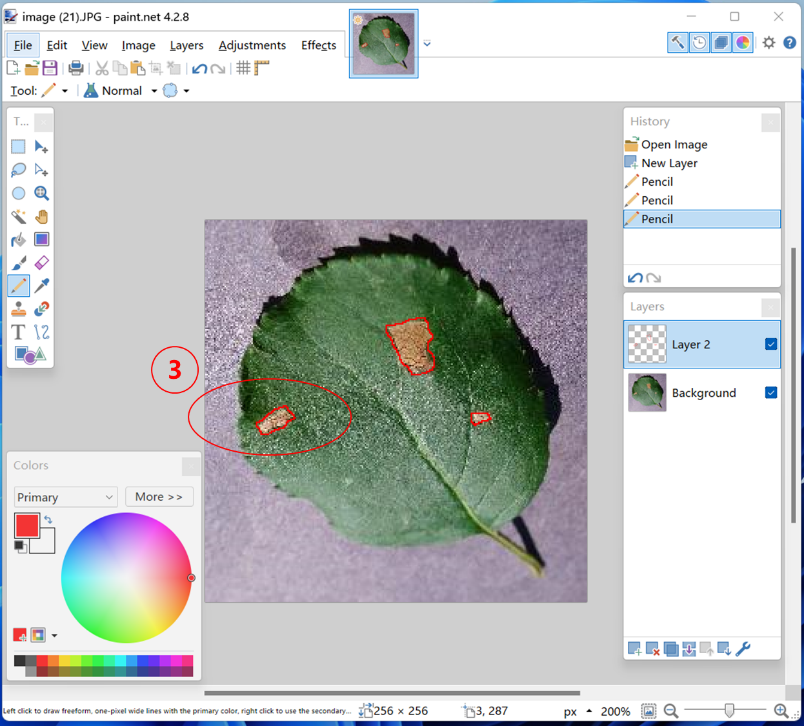

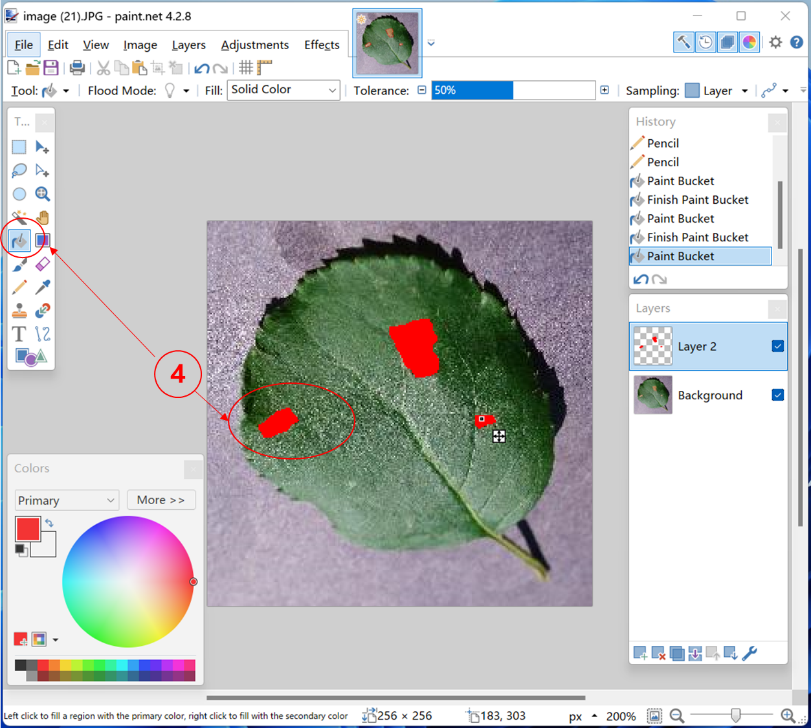


| (3) | (4) |
| --- | --- |


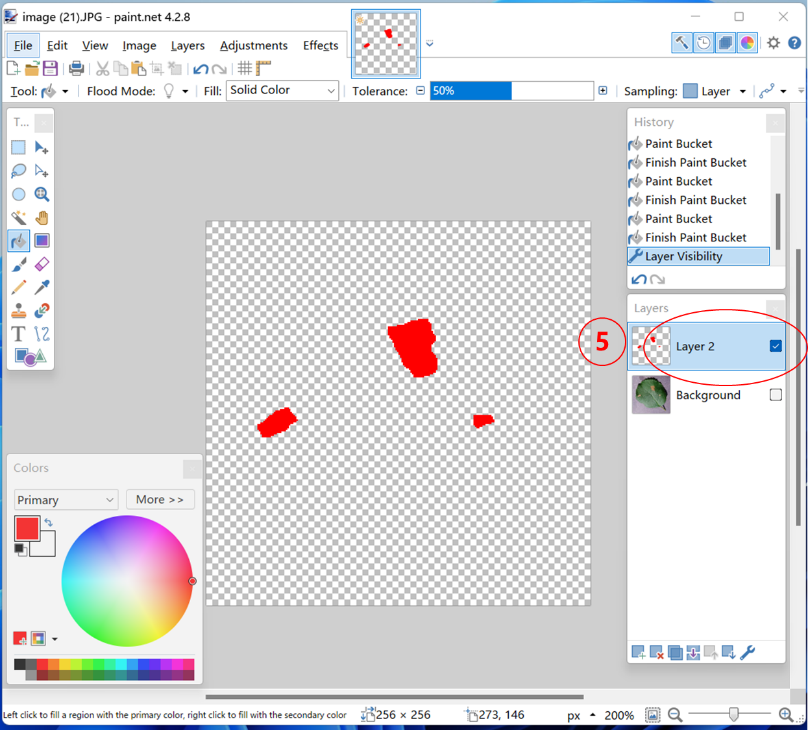


| (5) |  |
| --- | --- |

Supplement: Supplementary Materials — Figs. S1 to S5 [file plantphenomics.0022.f1.docx]
